# Efficient Oxidative Dearomatisations of Substituted Phenols Using Hypervalent Iodine (III) Reagents and Antiprotozoal Evaluation of the Resulting Cyclohexadienones against *T. b. rhodesiense* and *P. falciparum* Strain NF54

**DOI:** 10.3390/molecules27196559

**Published:** 2022-10-04

**Authors:** Nina Scheiber, Gregor Blaser, Eva-Maria Pferschy-Wenzig, Marcel Kaiser, Pascal Mäser, Armin Presser

**Affiliations:** 1 Institute of Pharmaceutical Sciences, Pharmaceutical Chemistry, University of Graz, Schubertstrasse 1, 8010 Graz, Austria; 2 Institute of Pharmaceutical Sciences, Pharmacognosy, University of Graz, Beethovenstrasse 8, 8010 Graz, Austria; 3 Swiss Tropical and Public Health Institute, Kreuzstrasse 2, 4123 Allschwil, Switzerland; 4 Swiss Tropical and Public Health Institute, University of Basel, Petersplatz 1, 4001 Basel, Switzerland

**Keywords:** oxidative dearomatization, hypervalent iodine, cyclohexadienones, antiprotozoal activity, physicochemical parameters

## Abstract

Quinones and quinols are secondary metabolites of higher plants that are associated with many biological activities. The oxidative dearomatization of phenols induced by hypervalent iodine(III) reagents has proven to be a very useful synthetic approach for the preparation of these compounds, which are also widely used in organic synthesis and medicinal chemistry. Starting from several substituted phenols and naphthols, a series of cyclohexadienone and naphthoquinone derivatives were synthesized using different hypervalent iodine(III) reagents and evaluated for their in vitro antiprotozoal activity. Antiprotozoal activity was assessed against *Plasmodium falciparum* NF54 and *Trypanosoma brucei rhodesiense* STIB900. Cytotoxicity of all compounds towards L6 cells was evaluated and the respective selectivity indices (SI) were calculated. We found that benzyl naphthoquinone **5c** was the most active and selective molecule against *T. brucei rhodesiense* (IC_50_ = 0.08 μM, SI = 275). Furthermore, the antiprotozoal assays revealed no specific effects. In addition, some key physicochemical parameters of the synthesised compounds were calculated.

## 1. Introduction

*p*-Quinols and quinones are cyclohexadienones and represent an important skeleton isolated from a variety of natural sources (bacteria, fungi, higher plants). Quinone derivatives are reported to exhibit a broad spectrum of biological activities such as anti-inflammatory [1,2], anticancer [3,4,5], antiviral [6], antitubercular [7,8], antifungal [9], antibacterial [2,7,10], and antiprotozoal [5,11,12], etc. Furthermore, the quinone and quinol moieties exhibit a strong preference for proteins containing cysteine thiols and serve therefore as potential covalent inhibitors of various biochemical targets [13,14].

As part of a program directed at the discovery of anticancer and antiprotozoal agents, we were interested in the synthesis of substituted naphthoquinones (NQs) and 4-hydroxycyclohexa-2,5-dien-1-ones because of their remarkable pharmacological activities [12,15,16,17,18,19]. Many methods are described in the literature for the oxidation of phenols to quinols and quinones [20,21,22,23,24], but most of them suffer from certain drawbacks, such as poor product selectivity or high toxicity of the catalysts.

**Figure 1:**
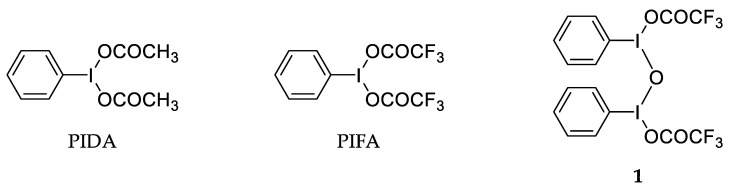
PIDA, PIFA, and the *μ*-oxo-bridged dimer **1**.

For our purpose, hypervalent iodine (III) reagents like diacetoxy iodobenzene (PhI(OAc)_2_, PIDA), bis(trifluoroacetoxy) iodo benzene (PhI(OCOCF_3_)_2_, PIFA) and the *μ*-oxo-bridged phenyl iodine trifluoroacetate **1** (Figure 1) evolved as reagents of choice. These reagents have been extensively used in organic synthesis [25,26,27]. The continued interest in hypervalent iodine species has led to the development of several chiral hypervalent iodine reagents and catalysts [28,29,30,31].

In this paper we report on the general preparation of *p*-quinols and naphthoquinones via a hypervalent iodine(III) mediated oxidation of the corresponding phenolic starting products together with our findings on the antiprotozoal activity and cytotoxicity towards L6 cells of the synthesised cyclohexadienones.

## 2. Results and Discussion

### 2.1. Chemistry

**Scheme 1:**
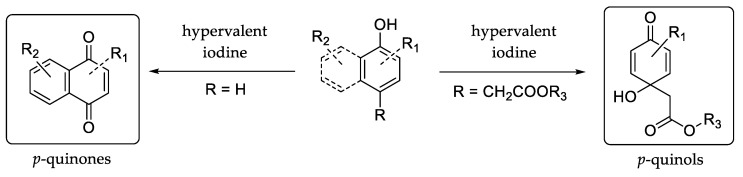
Phenolic oxidation using hypervalent iodine(III) reagents.

*p*-Benzoquinones and *p*-quinols can both be prepared from phenols by oxidative dearomatization [25,27]; however, a *p*-substituted phenol and water as a nucleophile is necessary for the synthesis of the 4-hydroxy quinol framework (Scheme 1). Among all environmentally friendly and non-metallic organic oxidants, hypervalent iodine reagents represent one of the most promising tools for the oxidative dearomatization of phenolic compounds [26,32,33].

Although well established and widely used, the mechanism of this reaction is still unclear and various possibilities of this process are discussed [28,34].

We have evaluated the three most common hypervalent iodine compounds: PIDA, PIFA and *μ*-oxo-bridged phenyl iodine trifluoroacetate **1** for the construction of *p*-quinones and *p*-quinols. As model substances, we used the commercially available starting materials methyl 4-hydroxyphenylacetate (**2a**) for the synthesis of the *p*-quinols and 1-naphthol (**3a**) for the design of NQ-derivatives (Scheme 1).

First of all, we chose the already known conversion of phenols into 4-substituted 4-hydroxy-cyclohexa-2,5-dienones with PIDA [35,36,37], PIFA [38,39] and the *μ*-oxo dimer **1** [40] and compared the yields of the obtained *p*-quinols. In addition, we also evaluated the reactivity of the hypervalent iodine PIFA in combination with the stable radical oxidant TEMPO [41].

Optimal conversion of the starting material was achieved by using oxidant **1** at 0 °C within only 10 min (Table 1). However, since the *μ*-oxo-dimer **1** is rather tricky to obtain, PIDA was also used as an alternative for all further conversions, as this reagent provided the second highest yields. We found that PIDA-mediated oxidation with about 20 min requires slightly more time for the complete conversion of the starting material. Hypervalent iodine(III)-mediated dearomatizations of a variety of phenols (**2a**–**i**) provided the corresponding quinols (**4a**–**i**) in usually good to moderate yields according to the optimized conditions (Table 1). The oxidation of the substrates **2j–l** failed and only led to decomposition products.

For the preparation of the NQ derivatives **5a**–**e** we used the long-established preparation from 1-naphthol [42] and compared the obtained yields of our available hypervalent iodine reagents (Table 1). For compound **3a** and **3b**, PIDA and *μ*-oxo dimer **1** provided similar yields within 90 min, but PIDA was ahead in the conversion of all other applied naphthols **3c**–**e**.

Dohi et al. reported an improved yield of **5a** when a larger amount of *μ*-oxo dimer **1** is applied [25]. We also made this observation, however, due to the laborious preparation of this oxidant (see experimental section), for the synthesis of our NQ derivatives the *μ*-oxo dimer **1** provides no advantages compared to PIDA.

### 2.2. Antiprotozoal Activity

The antiprotozoal activity of **5a**,**b** has already been described in the literature [43,44,45]. The synthesised *p*-quinols **4a**–**i** and *p*-quinones **5c**-**e** were now evaluated in vitro for their antiprotozoal activity against *P. falciparum* (NF54) and *T. brucei rhodesiense* (STIB900). Cytotoxicity was determined using L6 rat skeletal myoblasts to calculate a selectivity index for each parasite (SI = IC_50(L6)_/IC_50(parasite)_).

According to the recommended hit-to-lead identification criteria [46,47,48], all derivatives showed high activity towards NF54 (IC_50_ < 1 μM, Table 2), except **4g** and **4h**, which showed a moderate antiplasmodial effect (IC_50_ = 1–10 μM). However, the lack of selectivity of most compounds was disappointing in this series, and except for **4g** (SI = >12) and **5c** (SI = 24), all SI-values were in the single-digit ranges.

In contrast, promising trypanocidal activity was observed. According to the criteria set above, most of the tested derivatives showed high activity against *T. brucei rhodesiense*, and seven cyclhexadienones (**4b**–**f**, **5c**, **5d**) even showed an IC_50_ < 100 nM. The best results were found for the benzylnaphthoquinone **5c** with an IC_50_ of 80 nM and an SI of 275.

The ProTox-II data [49] of the tested compounds predicted low systemic and behavioral toxicity with LD_50_ values not exceeding 300 mg/kg. Therefore, these derivatives may have high potential for the development of new trypanocidal drugs [50].

### 2.3. Physicochemical Properties

Physicochemical parameters play a crucial role in drug development for the selection of potential drug candidates [51,52,53,54]. For this reason, an assessment of drug-likeness was made, and various physicochemical properties were calculated for all of the tested compounds (Table 2 and Supplementary Materials).

Almost all synthesized derivatives fulfil Lipinsky’s rule of five [55], Veber’s rule [56] and the drug-likeness classifier defined by Ghose et al. [57]; only compound **5e** failed in the Ghose filter.

It has been shown that log *D*_7.4_ (rather than log *P*) is one of the most significant physicochemical descriptors for optimizing permeability and solubility in drug development [58,59,60,61]. Accordingly, for all our synthesised cyclohexadienones, this parameter shows a certain correlation with the selectivity index (SI) of *P. falciparum* (R^2^ = 0.75) and *T.b. rhodesiense* (R^2^ = 0.80).

The use of ligand efficiency as a metric can also greatly simplify multi-parameter optimization in drug development [54,58,59,62]. The ligand efficiency metrics of our synthesised compounds (see the Supplementary Materials) closely agree with the values proposed for drug candidates, i.e., ligand efficiency (LE) > ~0.3, lipophilic ligand efficiency (LLE) > ~5, and lipophilicity-corrected ligand efficiency (LELP) −10 < LELP < 10 [63]. Furthermore, the observed selectivity indices and the calculated ligand efficiency metrics were strongly correlated (e.g., LLE*_P.f._*, R^2^ = 0.91; LELP*_T.b.r_*_._, R^2^ = 0.93).

## 3. Materials and Methods

### 3.1. Chemistry

#### 3.1.1. General Information

All reagents and solvents were purchased from Merck and Fluorochem Ltd. (Glossop, UK) The *μ*-oxo hypervalent iodine compound **1** was prepared from PhI(OCOCF_3_)_2_ (PIFA) according to the literature procedure [64].

The moisture-sensitive reactions were carried out under an inert argon atmosphere. Each reaction was observed by TLC on Merck TLC plates (silica gel 60 F254 0.2 mm, 200 × 200 mm) and detected at 254 nm. All reaction products were purified by flash column chromatography using silica gel 60 (Merck, 70–230 mesh, pore-diameter 60 Å), unless otherwise stated. Purity and homogeneity of the final compounds were assessed by TLC and high-resolution mass spectrometry. The melting points were determined with a digital melting point device (Electrothermal IA 9200).

The accurate structure elucidation was confirmed by 1D and 2D NMR spectroscopy on a Varian Unity Inova 400 MHz instrument (at 298 K) using 5 mm tubes. The chemical shifts are expressed in δ (ppm) using tetramethylsilane (TMS) as internal standard or the ^13^C signal of the solvent (CDCl_3_ δ 77.04 ppm). ^1^H NMR peak patterns are as follows: s (singlet), d (doublet), t (triplet), dd (double doublet), ddd (double dd), m (multiplet), br (broad singlet), coupling constants (J) were reported in Hertz (Hz). ^1^H and ^13^C resonances are numbered as given in the formulae (see Supplementary Materials); the signals marked with an asterisk are interchangeable.

High-resolution EI mass spectra (70 eV, source temperature 220 °C) were recorded on an orthogonal TOF spectrometer (Waters GCT Premier) equipped with a direct insertion (DI) probe. High resolution ESI and APCI mass spectra were acquired by analyzing sample solutions on an Ultimate 3000 HPLC hyphenated with a Q Exactive™ Hybrid Quadrupole-Orbitrap™ mass spectrometer equipped with a heated ESI II source or APCI source (Thermo Fisher Scientific), in the positive or negative ionization mode.

#### 3.1.2. General Procedure for the Dearomatization with *µ*-oxo dimer **1**. Method A:

The *µ*-oxo-bridged dimer (0.6 mmol) was added to a stirred solution of the corresponding phenol or naphthol (1 mmol) in CH_3_CN (6.50 mL) and H_2_O (2 mL) at 0 °C. The reaction mixture was stirred vigorously at 0 °C until the TLC showed complete consumption of the starting material (10–90 min). After the removal of CH_3_CN under reduced pressure, the resulting residue was extracted with CH_2_Cl_2_ several times. The combined organic layers were dried over Na_2_SO_4_ and concentrated in vacuo to give a residue, which was purified by flash chromatography.

*Methyl 2-(1-hydroxy-4-oxocyclohexa-2,5-dien-1-yl)acetate* (**4a**). Compound **4a** was obtained after stirring for 10 min and purified by flash chromatography using CHCl_3_/CH_3_CN (5:2). Colourless oil; yield 45%; *R*_f_ = 0.38 (CHCl_3_:CH_3_CN = 5:2). The spectroscopic data were found to be identical to the ones described in Ref [65]. Although **4a** represents an already known compound, to our knowledge the complete assignment of the NMR signals has not been published so far: ^1^H NMR (400 MHz, CDCl_3_): δ = 6.96 (d, *J* = 9.8 Hz, 2H, H-2, H-6), 6.21 (d, *J* =9.8 Hz, 2H, H-3, H-5), 3.98 (s, 1H, 1-OH), 3.76 (s, 3H, H-9), 2.71 (s, 2H, H-7) ppm; ^13^C NMR (100 MHz, CDCl_3_): δ = 184.9 (C-4), 171.3 (COOCH_3_), 148.8 (C-2, C-6), 128.3 (C-3, C-5), 67.3 (C-1), 52.3 (C-9), 43.3 (C-7) ppm.

*Methyl 2-(3-fluoro-1-hydroxy-4-oxocyclohexa-2,5-dien-1-yl)acetate* (**4b**). Compound **4b** was obtained after stirring for 10 min and purified by flash chromatography using CHCl_3_:CH_3_CN (3:1). Brownish oil; yield 32%; *R*_f_ = 0.53 (CHCl_3_:CH_3_CN = 3:1); ^1^H NMR (400 MHz, CDCl_3_): δ = 6.97 (dd, *J* = 10.1, 2.9 Hz, 1H, H-6), 6.54 (dd, ^3^*J*_H,F_ = 12.3 Hz, ^4^*J*_H,H_ = 2.9 Hz, 1H, H-2), 6.23 (dd, ^3^*J*_H,H_ = 10.1 Hz, ^4^*J*_H,F_ = 6.9 Hz, 1H, H-5), 4.06 (s, 1H, 1-OH), 3.78 (s, 3H, H-9), 2.80 (dd, *J* = 16.3, 1.3 Hz, 1H, H-7a), 2.74 (d, *J* = 16.3 Hz, 1H, H-7b) ppm; ^13^C NMR (100 MHz, CDCl_3_): δ = 177.8 (d, ^2^*J*_C,F_ = 22.6 Hz, C-4), 171.2 (C-8), 153.0 (d, ^1^*J*_C,F_ = 269.1 Hz, C-3), 149.5 (d, ^4^*J*_C,F_ = 2.5 Hz, C-6), 127.1 (d, ^3^*J*_C,F_ = 3.8 Hz, C-5), 124.4 (d, ^2^*J*_C,F_ = 12.0 Hz, C-2), 69.6 (d, ^3^*J*_C,F_ = 9.2 Hz, C-1), 52.5 (C-9), 43.3 (d, ^4^*J*_C,F_ = 2.5 Hz, C-7) ppm. HRMS (EI) calcd. for C_9_H_9_FO_4_ [M]^+^ = 200.0485; found: 200.0499.

*Methyl 2-(3-chloro-1-hydroxy-4-oxocyclohexa-2,5-dien-1-yl)acetate* (**4c**). Compound **4c** was obtained after stirring for 10 min and purified by flash chromatography using CH_2_Cl_2_/MeOH (25:1). Yellowish oil; yield 63%; *R*_f_ = 0.43 (CH_2_Cl_2_:MeOH = 25:1); ^1^H NMR (400 MHz, CDCl_3_: δ = 7.16 (s br, 1H, H-2), 6.98 (d br, *J* = 10.1 Hz, 1H, H-6), 6.30 (d, *J* = 10.1 Hz, 1H, H-5), 4.16 (s, 1H, 1-OH), 3.78 (s, 3H, H-9), 2.78 (d, *J* = 16.4 Hz, 1H, H-7a), 2.73 (d, *J* = 16.4 Hz, 1H, H-7b) ppm; ^13^C NMR (100 MHz, CDCl_3_): δ = 178.0 (C-4), 171.0 (C-8), 149.1 (C-6), 144.6 (C-2), 132.8 (C-3), 127.2 (C-5), 69.3 (C-1), 52.5 (C-9), 43.0 (C-7) ppm; HRMS (ESI) calcd. for C_9_H_10_ClO_4_ [M + H]^+^ = 217.0268; found: 217.0262.

*Methyl 2-(3-bromo-1-hydroxy-4-oxocyclohexa-2,5-dien-1-yl)acetate* (**4d**). Compound **4d** was obtained after stirring for 10 min and purified by flash chromatography using toluene/EtOAc (5:1). Yellow oil; yield 62%; *R*_f_ = 0.44 (toluene:EtOAc = 5:1); ^1^H NMR (400 MHz, CDCl_3_): δ = 7.43 (d, *J* = 2.9 Hz, 1H, H-2), 6.98 (dd, *J* = 10.0, 2.9 Hz, 1H, H-6), 6.31 (d, *J* = 10.0 Hz, 1H, H-5), 4.08 (s, 1H, 1-OH), 3.78 (s, 3H, H-9), 2.77 (d, *J* = 16.2 Hz, 1H, H-7a), 2.72 (d, *J* = 16.2 Hz, 1H, H-7b) ppm; ^13^C NMR (100 MHz, CDCl_3_): δ = 177.7 (C-4), 171.0 (C-8), 148.9 (C-2, C-6), 126.8 (C-5), 124.8 (C-3), 69.9 (C-1), 52.5 (C-9), 42.7 (C-7) ppm; HRMS (EI) calcd. for C_9_H_9_BrO_4_ [M]^+^ = 259.9684; found: 259.9693.

*Methyl 2-(3,5-dichloro-1-hydroxy-4-oxocyclohexa-2,5-dien-1-yl)acetate* (**4e**). Compound **4e** was obtained after stirring for 10 min and purified by flash chromatography using cyclohexane/EtOAc (2:1). Yellowish oil; yield 68%; *R*_f_ = 0.28 (cyclohexane:EtOAc = 2:1). The spectroscopic data were found to be identical to the ones described in Ref [66]. Although **4e** represents an already known compound, to our knowledge the complete assignment of the NMR signals has not been published so far: ^1^H NMR (400 MHz, CDCl_3_): δ = 7.15 (s, 1H, H-2, H-6), 4.14 (s, 1H, 1-OH), 3.80 (s, 3H, H-9), 2.78 (s, 2H, H-7) ppm; ^13^C NMR (100 MHz, CDCl_3_): δ = 172.2 (C-4), 170.9 (C-8), 144.5 (C-2, C-6), 132.0 (C-3, C-5), 69.7 (C-1), 52.6 (C-9), 42.7 (C-7) ppm; HRMS (EI) calcd. for C_9_H_8_Cl_2_O_4_ [M]^+^ = 249.9800; found: 249.9813.

*Methyl 2-(3,5-dibromo-1-hydroxy-4-oxocyclohexa-2,5-dien-1-yl)acetate* (**4f**). Compound **4f** was obtained after stirring for 10 min and purified by flash chromatography using toluene/EtOAc (5:1). Yellowish oil; yield 62%; *R*_f_ = 0.38 (toluene:EtOAc = 5:1). The spectroscopic data were found to be identical to the ones described in Ref [66]. Although **4f** represents an already known compound, to our knowledge the complete assignment of the NMR signals and also the HRMS data have yet to be published: ^1^H NMR (400 MHz, CDCl_3_): δ = 7.42 (s, 2H, H-2, H-6), 4.14 (s, 1H, 1-OH), 3.80 (s, 3H, H-9), 2.77 (s, 2H, H-7) ppm; ^13^C NMR (100 MHz, CDCl_3_): δ = 171.7 (C-4), 170.8 (C-8), 148.9 (C-2, C-6), 122.5 (C-3, C-5), 71.6 (C-1), 52.7 (C-9), 42.2 (C-7) ppm; HRMS (EI) calcd. for C_9_H_8_Br_2_O_4_ [M]^+^ = 337.8789; found: 337.8789.

*Methyl 3-(1-hydroxy-4-oxocyclohexa-2,5-dien-1-yl)propanoate* (**4g**). Compound **4g** was obtained after stirring for 20 min and purified by flash chromatography using CHCl_3_/CH_3_CN (5:2). Amber solid; yield 67%; *R*_f_ = 0.31 (CHCl_3_:CH_3_CN = 5:2); m.p.: 49-50 °C; ^1^H NMR (400 MHz, CDCl_3_): δ = 6.83 (d, *J* = 10.3 Hz, 2H, H-2, H-6), 6.20 (d, *J* = 10.3 Hz, 2H, H-3, H-5), 3.67 (s, 3H, H-10), 2.60 (s, 1H, 1-OH), 2.36 (t, *J* = 7.6 Hz, 2H, H-8), 2.12 (m, 2H, H-7) ppm; ^13^C NMR (100 MHz, CDCl_3_): δ = 185.1 (C4), 173.4 (C-9), 150.2 (C-2, C-6), 128.6 (C-3, C-5), 69.2 (C-1), 52.0 (C-10), 34.5 (C-7), 28.6 (C-8) ppm; HRMS (ESI) calcd. for C_10_H_13_O_4_ [M + H]^+^ = 197.0814; found: 197.0808.

*1-Oxaspiro [4.5]deca-6,9-diene-2,8-dione* (**4h**). Compound **4h** was obtained after stirring for 20 min and purified by flash chromatography using CHCl_3_/CH_3_CN (5:2). Amber solid; yield 8%; *R*_f_ = 0.54 (CHCl_3_:CH_3_CN = 5:2); m.p.: 105–106 °C (lit [67] m.p. 104–106 °C). The spectroscopic data were found to be identical to the ones described in Ref [67]. Although **4h** represents an already known compound, to our knowledge the complete assignment of the NMR signals has not been published so far: ^1^H NMR (400 MHz, CDCl_3_): δ = 6.86 (d, *J* = 10.1 Hz, 2H, H-2, 6), 6.29 (d, *J* = 10.1 Hz, 2H, H-3, 5), 2.79 (t, *J* = 8.3 Hz, 2H, H-8), 2.38 (t, *J* = 8.3 Hz, 2H, H-7) ppm; ^13^C NMR (100 MHz, CDCl_3_): δ = 184.1 (C-4), 175.1 (C-9), 145.5 (C-2, C-6), 129.2 (C-3, C-5), 78.4 (C-1), 32.3 (C-7), 28.0 (C-8) ppm.

*Methyl (2E)-3-(1-hydroxy-4-oxocyclohexa-2,5-dien-1-yl)prop-2-enoate* (**4i**). Compound **4i** was obtained after stirring for 10 min and purified by flash chromatography using CHCl_3_/CH_3_CN (7:1). Amber oil; yield 15%; *R*_f_ = 0.28 (CHCl_3_:CH_3_CN = 7:1). The spectroscopic data were found to be identical to the ones described in Ref [68]. Although **4i** represents an already known compound, to our knowledge the complete assignment of the NMR signals has not been published so far: ^1^H NMR (400 MHz, CDCl_3_): δ = 6.76 (d, *J* = 10.1 Hz, 2H, H-2, H-6), 6.67 (d, *J* = 15.5 Hz, 1H, H-7), 6.29 (d, *J* = 15.5 Hz, 1H, H-8), 6.24 (d, *J* = 10.1 Hz, 2H, H-3, H-5), 3.76 (s, 3H, H-10) ppm; ^13^C NMR (100 MHz, CDCl_3_): δ = 184.9 (C-4), 166.3 (C-9), 148.1 (C-2, C-6), 145.1 (C-7), 128.4 (C-3, C-5), 122.3 (C-8), 69.6 (C-1), 52.0 (C-10) ppm.

*2-Methyl-1,4-dihydronaphthalene-1,4-dione* (**5b**). Compound **5b** was obtained after stirring for 90 min and purified by flash chromatography using CHCl_3_/cyclohexane (6:1). Amber solid; yield: 73%; *R*_f_ = 0.45 (CHCl_3_:cyclohexane = 6:1); m.p.: 107–108°C (lit [69] m.p. 106–107 °C). The analytical data agreed with the literature [69].

#### 3.1.3. General Procedure for the Dearomatisation with PIDA. Method B:

Diacetoxy iodobenzene (PIDA) (1.30 equiv.) was added in small portions to a stirred solution of the corresponding phenol or naphthol (1 equiv.) in CH_3_CN (6.5 mL) and H_2_O (2 mL) at 0 °C. The reaction mixture was allowed to reach ambient temperature and stirred vigorously until the TLC showed complete consumption of the starting material (20–150 min). The orange coloured mixture was diluted with sat. aq NaHCO_3_ and then extracted with EtOAc several times. The combined organic extracts were washed with brine, dried over Na_2_SO_4_ and concentrated in vacuo to give a residue, which was purified by flash chromatography.

*1,4-Dihydronaphthalene-1,4-dione* (**5a**). Compound **5a** was obtained after stirring for 150 min and purified by flash chromatography using CHCl_3_/cyclohexane (6:1). Amber solid; yield: 76%; *R*_f_ = 0.64 (CHCl_3_:cyclohexane = 6:1); m.p.: 123–125° (lit [70] m.p. 124-125 °C). The analytical data agreed with the literature [71].

*2-Benzyl-1,4-dihydronaphthalene-1,4-dione* (**5c**). Compound **5c** was obtained after stirring for 90 min and purified by flash chromatography using cyclohexane/EtOAc (8.5:1.5). Ochre solid; yield: 82%; *R*_f_ = 0.58 (cyclohexane:EtOAc = 8.5:1.5); m.p.: 92–93 °C (lit [72] m.p. 93–94 °C). The spectroscopic data were found to be identical to the ones described in [72]. Although **5c** represents an already known compound, to our knowledge the complete assignment of the NMR signals has not been published so far: ^1^H NMR (400 MHz, CDCl_3_): δ = 8.11 (m, 1H, H-8), 8.04 (m, 1H, H-5), 7.72 (m, 2H, H-6, H-7), 7.34 (m, 2H, H-3′, H-5′), 7.26 (m, 1H, H-4′), 7.25 (m, 2H, H-2′, H-6′), 6.61 (t, J = 1.5 Hz, 1H, H-3), 3.90 (m, 2H, CH_2_-Bn) ppm; ^13^C NMR (100 MHz, CDCl_3_): δ = 185.2 (C-4), 185.0 (C-1), 150.9 (C-2), 136.7 (C-1′), 135.6 (C-3), 133.8* (C-6, C-7), 133.7* (C-6, C-7), 132.2 (C-8a), 132.1 (C-4a), 129.4 (C-2′, C-6′), 128.9 (C-3′, C-5′), 127.0 (C-4′), 126.7 (C-8), 126.1 (C-5), 35.7 (CH_2_-Bn) ppm; HRMS (EI) calcd. for C_17_H_12_O_2_ [M]^+^ = 248.0837; found: 248.0837.

*6-Fluoro-1,4-dihydronaphthalene-1,4-dione* (**5d**). Compound **5d** was obtained after stirring for 90 min and purified by flash chromatography using cyclohexane/EtOAc (3:1). Amber solid; yield: 87%; *R*_f_ = 0.46 (cyclohexane:EtOAc = 3:1); m.p.: 119–120 °C (lit [73] m.p. 119.8–121.4 °C). The spectroscopic data were found to be identical to the ones described in Ref [73]. Although **5d** represents an already known compound, to our knowledge the complete assignment of the NMR signals have not been published so far: ^1^H NMR (400 MHz, CDCl_3_): δ = 8.14 (dd, ^3^*J*_H,H_ = 8.6, ^4^*J*_H,F_ = 5.2 Hz, 1H, H-5), 7.73 (dd, ^3^*J*_H,F_ = 8.5, ^4^*J*_H,H_ = 2.6 Hz, 1H, H-8), 7.43 (td, ^3^*J*_H,F_ = 8.5, ^3^*J*_H,H_ = 8.5, ^4^*J*_H,H_ = 2.6 Hz, 1H, H-6), 7.02 (d, *J* = 10.4 Hz, 1H, H-2), 6.99 (d, *J* = 10.4 Hz, 1H, H-3) ppm; ^13^C NMR (100 MHz, CDCl_3_): δ = 183.9 (d, ^4^*J*_C,F_ = 1.5 Hz, C-1), 183.6 (d, ^5^*J*_C,F_ = 1.0 Hz, C-4), 166.1 (d, ^1^*J*_C,F_ = 257.6 Hz, C-7), 138.9 (C-3), 138.6 (d, ^5^*J*_C,F_ = 2.0 Hz, C-2), 134.5 (d, *^3^J_C_*_,F_ = 8.0 Hz, C-8a), 129.8 (d, ^3^*J*_C,F_ = 9.0 Hz, C-5), 128.5 (d, ^4^*J*_C,F_ = 3.3 Hz, C-4a), 121.2 (d, ^2^*J*_C,F_ = 22.6 Hz, C-6), 113.2 (d, ^2^*J*_C,F_ = 23.6 Hz, C-8) ppm.

*6,7-Difluoro-1,4-dihydronaphthalene-1,4-dione* (**5e**). Compound **5e** was obtained after stirring for 150 min at room temperature and purified by flash chromatography using cyclohexane/EtOAc (3:1). Amber solid; yield: 82%; *R*_f_ = 0.50 (cyclohexane:EtOAc = 3:1); mp: 128–130 °C. With the exception of the melting point, the compound was described as oil in the reference, and all spectroscopic data are identical to those given in Ref [74]. Although **5e** represents an already known compound, to our knowledge the complete assignment of the NMR signals has not been published so far: ^1^H NMR (400 MHz, CDCl_3_): δ = 7.89 (t, ^3^*J*_H,F_ = 8.6 Hz, ^4^*J*_H,F_ = 8.6 Hz, 2H, H-5, H-8), 7.01 (s, 2H, H-2, H-3) ppm; ^13^C NMR (100 MHz, CDCl_3_): δ = 182.7 (C-1, C-4), 154.1 (dd, ^1^*J*_C,F_ = 262.2, ^2^*J*_C,F_ = 15.1 Hz, C-6, C-7), 138.8 (C-2, C-3), 129.8 (t, ^3^*J*_C,F_ = 4.9, ^4^*J*_C,F_ = 4.9 Hz, C-4a, C-8a), 116.0 (dd, ^2^*J*_C,F_ = 13.4, ^3^*J*_C,F_ = 7.5 Hz, C-5, C-8) ppm; HRMS (APCI) calcd. for C_10_H_4_F_2_O_2_ [M]^−^ = 194.0179; found: 194.0183.

### 3.2. Biological Testing

#### 3.2.1. Assay for In Vitro Antimalarial Activity

Antimalarial activity was determined in vitro against the erythrocytic stages of *P. falciparum* using the drug-sensitive strain NF54. Parasite proliferation was assessed by incorporation of [^3^H]-hypoxanthine using a modified version of [75]; for details, please refer to the Supplementary Materials. The positive control was chloroquine.

#### 3.2.2. Assay for In Vitro Trypanocidal Activity

Trypanocidal activity was determined in vitro against axenically grown bloodstream-forms of *T. b. rhodesiense* STIB900 as described in Refs. [76,77] and detailed in the Supplementary Materials. Parasite proliferation was assessed via fluorescence of the redox-sensitive dye resazurin (Alamar blue). The drug melarsoprol was used as the positive control.

#### 3.2.3. Assay for Cytotoxicity

Cytotoxicity was determined in vitro against rat L6 myoblasts as described [78]; details are listed in the supplement. Cell proliferation was assessed with resazurin, and the generally cytotoxic agent podophyllotoxin served as the positive control.

## 4. Conclusions

The *μ*-oxo-dimer **1** can be used instead of PIDA and PIFA for oxidative dearomatizations in aqueous media., This reagent provided the highest yields, especially in the conversion of phenols into substituted 4-hydroxy-cyclohexa-2,5-dienones. However, the laborious preparation prevents a general use of this oxidant.

Many of the synthesised compounds showed promising biological activities (Table 2) as well as favourable physicochemical properties. The trypanocidal effects were extraordinary, and remarkable selectivity could be achieved, especially in the case of **5c** (IC_50_ = 0.08 μM; SI = 275). In contrast, assays on antiprotozoal activity revealed high activity but no specific effects. The results presented in this paper demonstrate the potential of quinols and quinones for the development of new anti-infectives and identify PIDA as the most probable reagent for the preparation of these valuable compounds

## Figures and Tables

**Table 1:** Oxidation of phenols (**2a**–**l**) and naphthols (**3a**–**e**) in aqueous CH_3_CN using PIDA, PIFA and the *μ*-oxo dimer **1**.

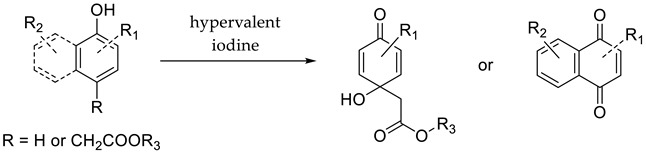					
Entry	Substrate	Product	Method ^a^	Time ^b^	Yield/% ^c^
1	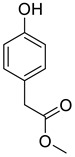 **2a**	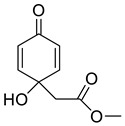 **4a**	A B C D	10 20 30 30	45 42 39 34
2	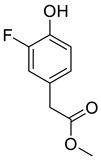 **2b**	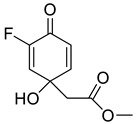 **4b**	A B	10 20	32 29
3	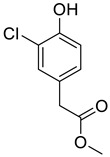 **2c**	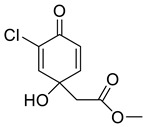 **4c**	A B	10 20	63 53
4	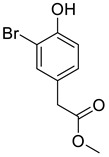 **2d**	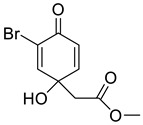 **4d**	A B	10 20	62 41
5	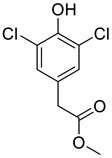 **2e**	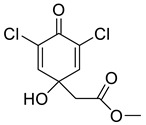 **4e**	A B	10 20	68 47
6	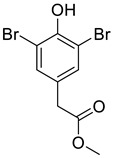 **2f**	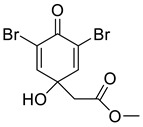 **4f**	A B	10 20	62 34
7	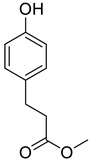 **2g**	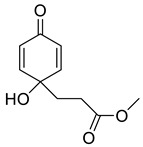 **4g**	A B	20 20	67 60
8	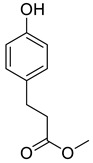 **2h**	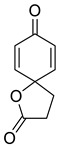 **4h**	A B	20 20	8 6
9	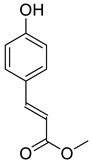 **2i**	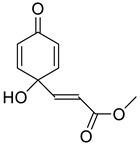 **4i**	A B	10 20	15 11
10	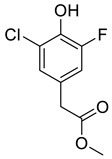 **2j**	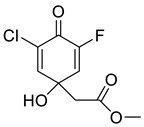 **4j**	A B	10 20	0 0
11	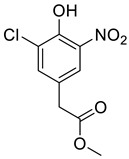 **2k**	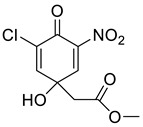 **4k**	A B	10 20	0 0
12	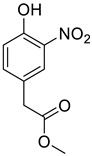 **2l**	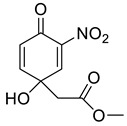 **4l**	A B	10 20	0 0
13	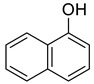 **3a**	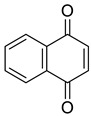 **5a**	A B C D	90 90 60 90	75 76 28 46
14	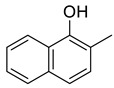 **3b**	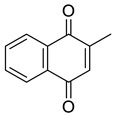 **5b**	A B	90 90	73 71
15	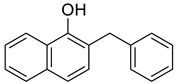 **3c**	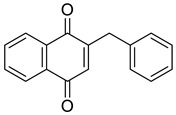 **5c**	A B	90 90	65 82
16	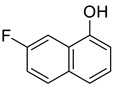 **3d**	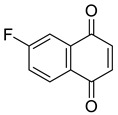 **5d**	A B	90 90	74 87
17	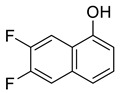 **3e**	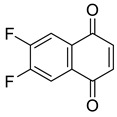 **5e**	A B	90 150	71 82

^a^ Reagents and conditions: phenolic substrates (**2a**–**l**, **3a**–**e**); method A: *μ*-oxo dimer **1**, CH_3_CN/H_2_O), 0 °C; method B: PhI(OAc)_2_, CH_3_CN/H_2_O, 0 °C (quinols), 0 °C → RT (quinones); method C: PhI(OCOCF_3_)_2_, CH_3_CN/H_2_O, 0 °C; method D: PhI(OCOCF_3_)_2_, TEMPO, CH_3_CN/H_2_O, RT. ^b^ Reaction time (min); ^c^ Isolated yield.

**Table 2:** In vitro antiparasitic activity, host toxicity and key physicochemical properties of the tested compounds.

ID No.	*P. falc.* ^a^	SI ^b^	*T. b. rhod*. ^c^	SI ^b^	Cyt. L6 ^d^	Chemical	log *P*	log *D_7_*_.4_
	IC_50_ μM		IC_50_ μM		IC_50_ μM	Structure		
Chl.	0.002	45,500			91.1			
Mel.			0.004	6050	24.2			
Pod.					0.007			
**4a**	0.969	0.40	0.588	0.66	0.391	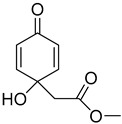	0.11	0.19
**4b**	0.644	1.02	0.078	8.42	0.657	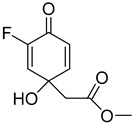	−0.37	0.12
**4c**	0.837	0.53	0.047	9.36	0.440	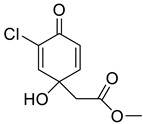	0.01	0.58
**4d**	0.301	0.71	0.042	5.09	0.214	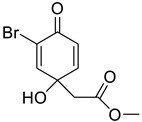	0.28	0.75
**4e**	0.245	0.47	0.01	11.6	0.116	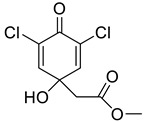	−0.09	0.97
**4f**	0.391	0.51	0.024	8.29	0.199	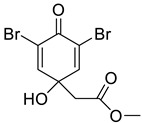	0.46	1.30
**4g**	8.16	>12.3	4.87	>20.5	>100	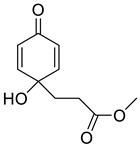	0.36	0.48
**4h**	2.75	0.15	4.89	0.09	0.425	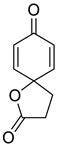	0.93	1.00
**4i**	0.657	0.87	0.108	5.27	0.569	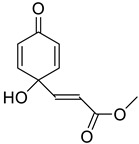	1.28	0.87
**5c**	0.917	23.97	0.08	274.75	21.98	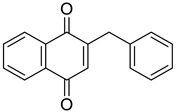	2.93	3.47
**5d**	0.677	3.21	0.093	23.33	2.17	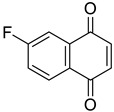	1.18	1.64
**5e**	0.689	6.97	0.166	28.92	4.80	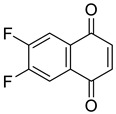	1.32	1.78

^a^*P. falciparum*, strain NF54, erythrocytic stages; ^b^ SI is defined as the ratio: IC_50_ in L6 cells/IC_50_ in each parasite; ^c^ *T. brucei rhodesiense*, strain STIB900 trypomastigote forms; ^d^ cytotoxicity L6 cells rat skeletal myoblasts. Reference drugs: *P. falc*., chloroquine (chl.), *T. b. rhod*., melarsoprol (mel.), Cyt. L6, podophyllotoxin (pod.). The IC_50_ value of each reference drug is the mean from multiple measurements in parallel with the compounds of interest. The physical properties were predicted by using MarvinSketch 21.13.0, ChemAxon (https://www.chemaxon.com acceseed on 27 September 2022). IC_50_ values of the tested compounds are the means of two to three measurements. The SD was <5%.

## Data Availability

The data presented in this study are available on request from corresponding author.

## Supplementary Materials

The following supporting information can be downloaded at: https://www.mdpi.com/article/10.3390/molecules27196559/s1, Table S1: Calculated physicochemical properties of the tested compounds; Table S2: Calculated ligand efficiency metrics of the tested compounds; Experimental of biological testing; ^1^H- and ^13^C-NMR spectra of the prepared compounds.

## Sample Availability

Samples of the compounds are not available from the authors.
