# Supplementary material for: Efficient Oxidative Dearomatisations of Substituted Phenols Using Hypervalent Iodine (III) Reagents and Antiprotozoal Evaluation of the Resulting Cyclohexadienones against T. b. rhodesiense and P. falciparum Strain NF54
Source: Molecules. 2022 Oct 4;27(19):6559. doi: 10.3390/molecules27196559 (PMC9573667; doi:10.3390/molecules27196559)

Efficient Oxidative Dearomatisations of Substituted Phenols using hypervalent iodine (III) reagents and Antiprotozoal Evaluation of the resulting Cyclohexadienones against *T. b. rhodesiense* and *P. falciparum* strain NF54

Nina Scheiber[1] ● Gregor Blaser[1] ● Eva-Maria Pferschy-Wenzig[2] ● Marcel Kaiser[3,4] ● Pascal Mäser[3,4] ● Armin Presser[1]

1 Institute of Pharmaceutical Sciences, Pharmaceutical Chemistry, University of Graz, Schubertstrasse 1, A-8010 Graz, Austria

2 Institute of Pharmaceutical Sciences, Pharmacognosy, University of Graz, Beethovenstrasse 8, A-8010 Graz, Austria

3 Swiss Tropical and Public Health Institute, Kreuzstrasse 2, CH-4123 Allschwil, Switzerland

4 University of Basel, Petersplatz 1, CH-4001 Basel, Switzerland

Supporting Information

Table of contents

1. Experimental of biological testing **S2**

2. Calculated physicochemical parameters and models **S3-S4**

3. NMR spectra of the prepared compounds **S5-S16**

1. Experimental of biological testing

1.1 In vitro growth inhibition assay of Plasmodium falciparum NF54

Plasmodium falciparum, strain NF54, erythrocytic stages, and the standard drug, chloroquine, were used for the assay. The parasite cultures incubated in RPMI 1640 medium with 5% AlbuMAX™ (without hypoxanthine) were exposed to serial drug dilutions in microtiter plates. After 48 h of incubation at 37 °C in a reduced oxygen atmosphere, 0.5 μCi [^{3}H]-hypoxanthine was added to each well of the plate. Cultures were incubated for a further 24 h before they were harvested onto glass-fiber filters and washed with distilled water. The radioactivity was counted using a Betaplate™ liquid scintillation counter (Wallac, Zurich). The results were recorded as counts per minute (CPM) per well at each drug concentration and expressed as percentage of the untreated controls. IC_{50} values were calculated from the sigmoidal inhibition curves using Microsoft Excel. Chloroquine was used as control.

1.2 In vitro growth inhibition assay of Trypanosoma brucei rhodesiense

Trypanosoma brucei rhodesiense, STIB 900 strain, and the standard drug, melarsoprol, were used for the assay. Minimum Essential Medium (50 μL) supplemented with 25 mM HEPES, 1g/L additional glucose, 1% MEM non-essential amino acids (100x), 0.2 mM 2-mercaptoethanol, 1 mM Na-pyruvate and 15% heat-inactivated horse serum was added to each well of a 96-well microtiter plate. Serial drug dilutions of 11 three-fold dilution steps covering a range from 100 to 0.002 μg/mL were prepared. Then 4×10^3 bloodstream forms of *T. b. rhodesiense* (STIB 900) in 50 μL were added to each well and the plate incubated at 37 °C under a 5% CO_2 atmosphere for 72 h. 10 μL Alamar Blue (resazurin, 12.5 mg in 100 mL double-distilled water) was then added to each well and incubation continued for a further 2–4 h. Then, the plates were read with a Spectramax Gemini XS microplate fluorometer (Molecular Devices Cooperation, Sunnyvale, CA, USA) using an excitation wavelength of 536 nm and an emission wavelength of 588 nm. The IC_{50} values were calculated from the sigmoidal inhibition curves using the microplate reader software Softmax Pro (Molecular Devices Cooperation, Sunnyvale, CA, USA). Melarsoprol was used as control.

1.3 Cytotoxicity against L6 cells

Assays were performed in 96-well microtiter plates, each well containing 100 μL of RPMI 1640 medium supplemented with 1% L-glutamine (200 mM) and 10% foetal bovine serum, and 4000 L6 cells (a primary cell line derived from rat skeletal myoblasts). Serial drug dilutions of 11 threefold dilution steps covering a range from 100 to 0.002 μg/mL were prepared. After 72 h of incubation, the plates were inspected under an inverted microscope to assure growth of the controls and sterile conditions. 10 μL of Alamar Blue solution was then added to each well and the plates incubated for another 2 h. Then the plates were read with a Spectramax Gemini XS microplate fluorometer (Molecular Devices Cooperation, Sunnyvale, CA, USA) using an excitation wavelength of 536 nm and an emission wavelength of 588 nm. The IC_{50} values were calculated by linear regression from the sigmoidal dose inhibition curves using the microplate reader software Softmax Pro (Molecular Devices Cooperation, Sunnyvale, CA, USA). Podophyllotoxin (Sigma P4405) was used as control.

2. Calculated physicochemical parameters and models

Table S1: Calculated physicochemical properties of the tested compounds.

compd	MW	log*S*	MR	mPol (Å³)	vdWVol (Å³)	tPSA (Å²)	ASA (Å²)	ASA*pho* (Å²)	ASA*pol* (Å²)
						pH 7.4	pH 7.4	pH 7.4	pH 7.4
**4a**	182.18	-1.56	47.24	17.12	159.81	63.6	313.60	216.76	96.84
**4b**	200.17	-1.77	47.44	17.21	164.72	63.6	321.47	223.64	97.83
**4c**	216.62	-2.10	52.03	19.28	173.81	63.6	329.03	230.70	98.32
**4d**	261.07	-2.23	54.85	20.47	177.94	63.6	333.60	235.68	97.92
**4e**	251.06	-2.63	56.82	21.44	187.69	63.6	342.37	243.79	98.58
**4f**	339.97	-2.81	62.46	23.75	196.27	63.6	350.86	251.60	99.26
**4g**	196.20	-1.49	51.99	19.31	176.90	63.6	347.75	242.96	104.79
**4h**	164.16	-2.11	43.73	15.70	139.52	43.37	266.87	179.18	87.68
**4i**	194.19	-2.24	52.93	18.75	169.14	63.6	333.31	218.02	115.29
**5c**	248.28	-4.72	75.24	26.25	222.93	34.14	416.26	359.88	56.38
**5d**	176.15	-3.39	46.40	15.55	140.15	34.14	284.39	154.13	130.26
**5e**	194.14	-3.69	46.62	15.66	145.02	34.14	290.81	170.82	119.98

The molecular weight (MW), log*S*, molar refractivity (MR), molecular polarizability (mPol), van der Waals volume (vdWVol), topological polar surface area (tPSA), solvent accessible surface area (ASA), hydrophobic accessible surface area (ASA*pho*) and polar accessible surface area (ASA*pol*) were calculated using Marvin 21.13.0, ChemAxon (https://www.chemaxon.com).

Table S2: Calculated ligand efficiency metrics of the tested compounds.

compd	*P. falciparum*			*T. brucei rhodesiense*		
	LE	LLE	LELP	LE	LLE	LELP
**4a**	0.63462	6.1690	-0.24471	0.65751	6.3859	-0.23619
**4b**	0.60668	6.1451	0.07582	0.69651	7.0619	0,06604
**4c**	0.59552	5.8133	0.44331	0.71807	7.0639	0.36765
**4d**	0.63904	6.1264	0.61811	0.72286	6.9818	0.54644
**4e**	0.60462	5.9275	1.1301	0.73167	7.3167	0.93389
**4f**	0.58605	5.4625	1.613	0.6969	6.6745	1.3564
**4g**	0.49861	4.7892	0.59987	0.52058	5.0134	0.57456
**4h**	0.63571	5.4846	0.11971	0.60714	5.2346	0.12534
**4i**	0.60583	6.1355	0.07742	0.68266	6,9197	0.06870
**5c**	0.43594	2.7161	7.6191	0.51243	3.7754	6.4819
**5d**	0.65105	4.6292	2.3657	0.74203	5.4913	2.0757
**5e**	0.60380	4.5208	2.7178	0.66437	5.1389	2.470

The ligand efficiency (LE), lipophilic ligand efficiency (LLE) and ligand efficiency lipophilic price (LELP) are based on IC_{50} values in nmol/L and were calculated using the DataWarrior software, version 5.5.0 (http://www.openmolecules.org/datawarrior.html).

3. NMR spectra of the prepared compounds

Methyl 2-(1-hydroxy-4-oxocyclohexa-2,5-dien-1-yl)acetate (**4a**)

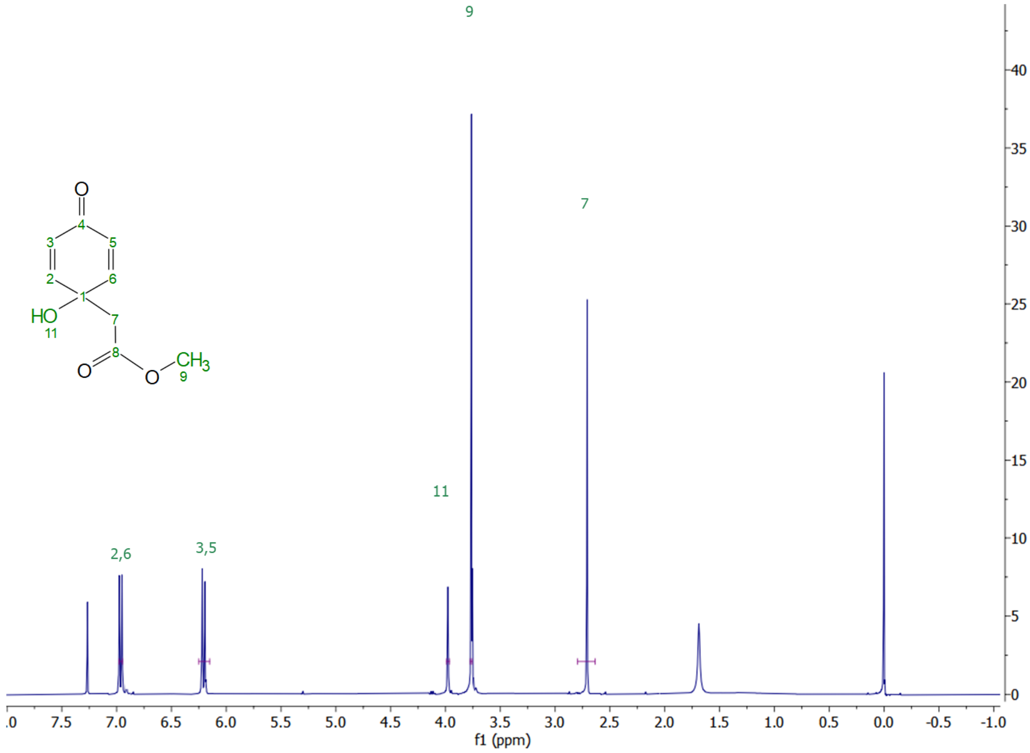

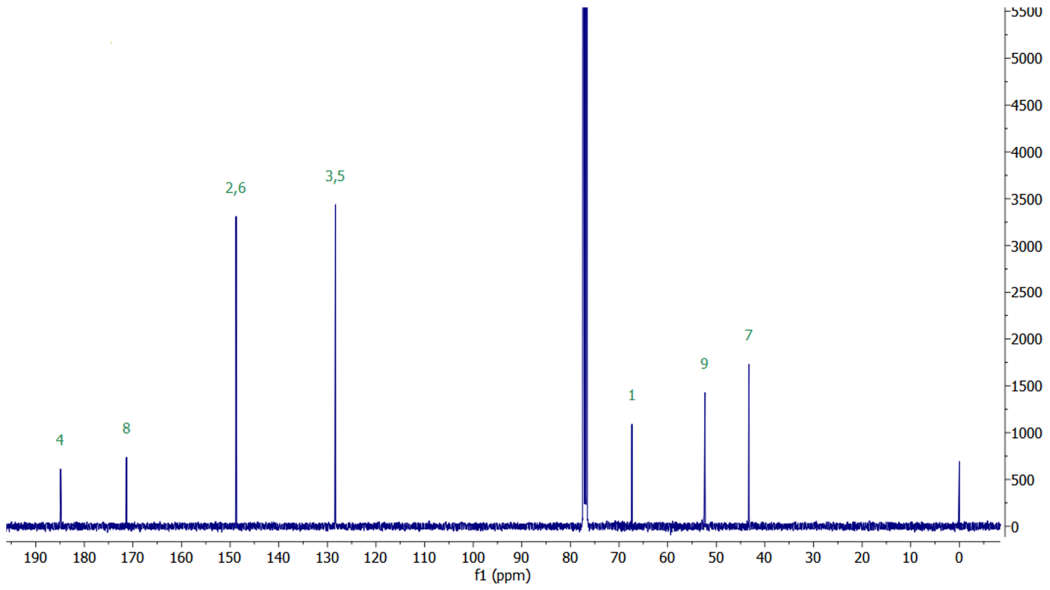

Methyl 2-(3-fluoro-1-hydroxy-4-oxocyclohexa-2,5-dien-1-yl)acetate (**4b**)

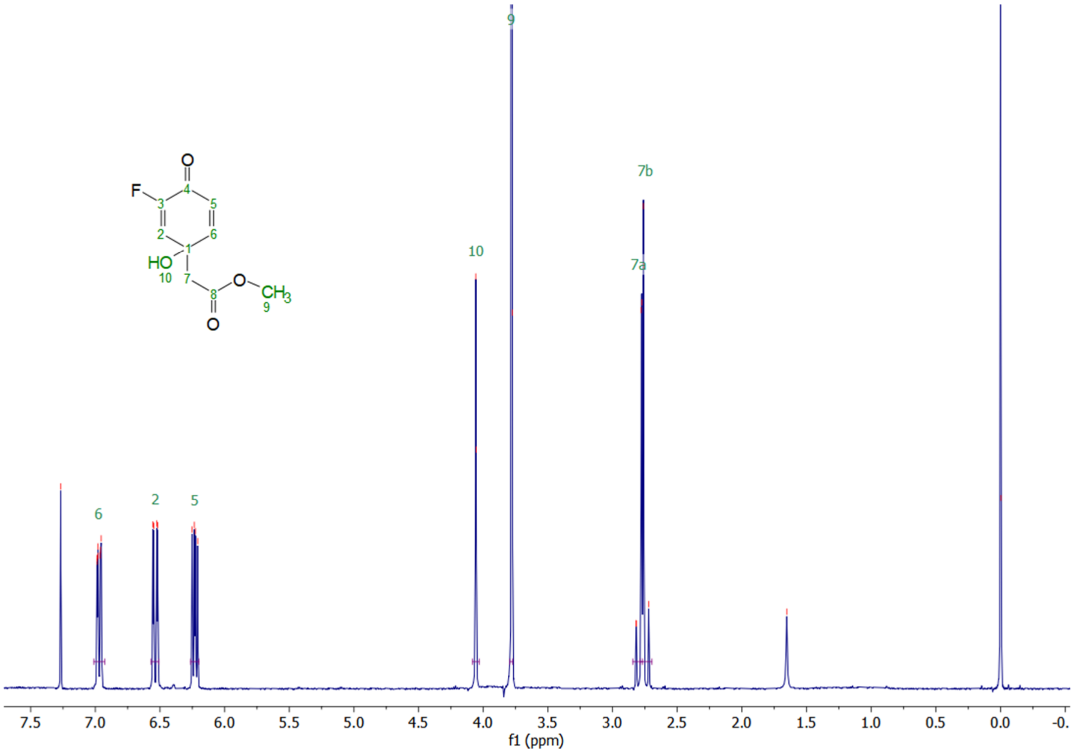

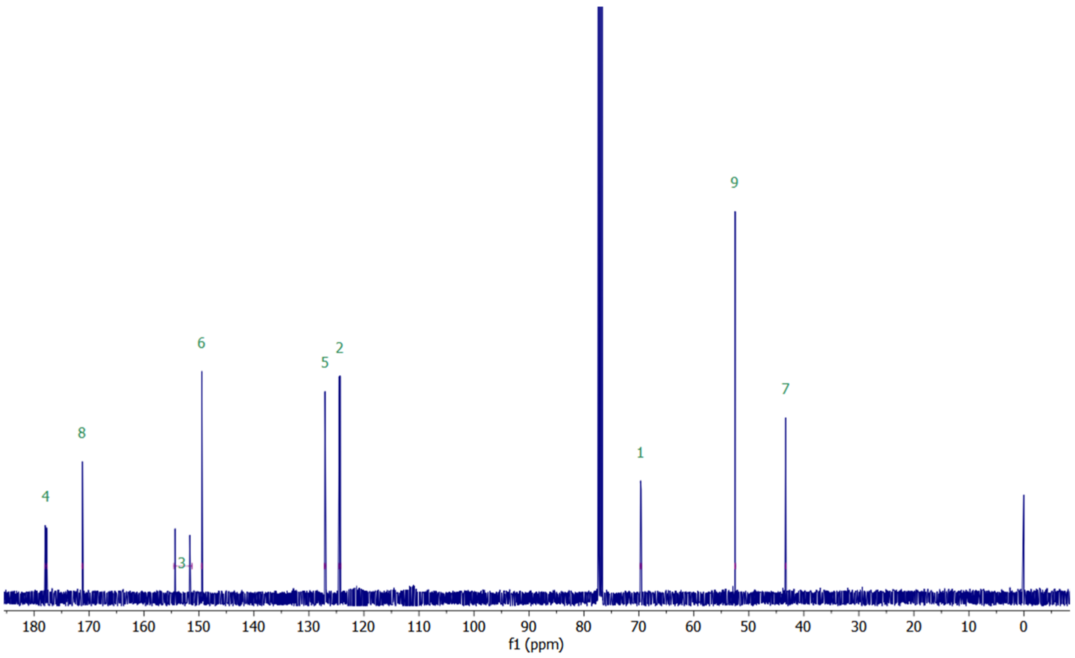

Methyl 2-(3-chloro-1-hydroxy-4-oxocyclohexa-2,5-dien-1-yl)acetate (**4c**)

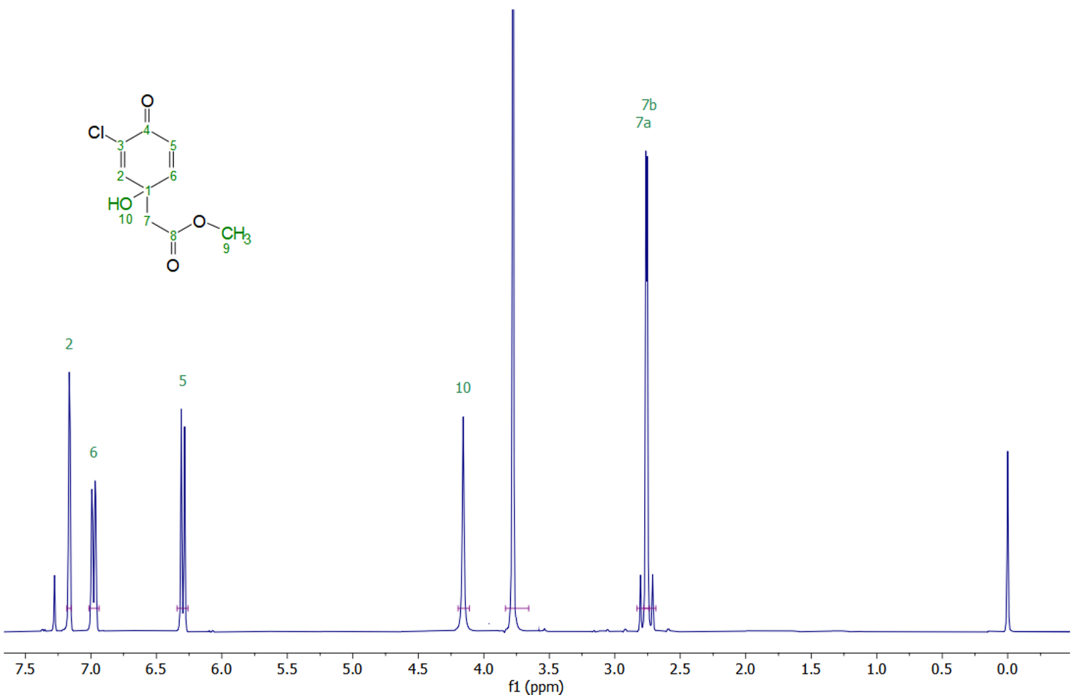

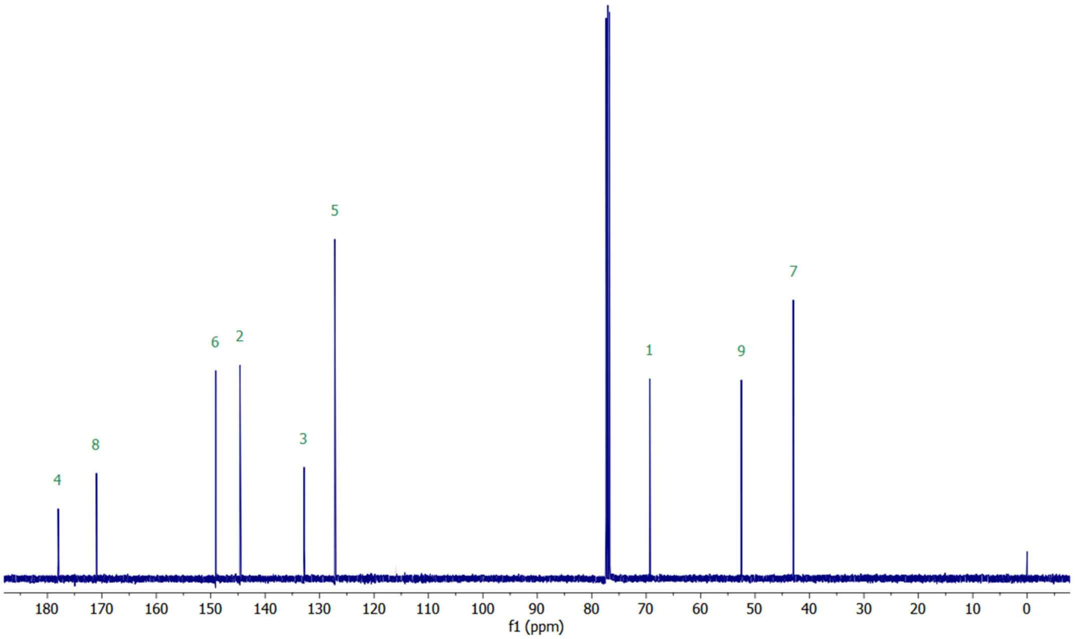

Methyl 2-(3-bromo-1-hydroxy-4-oxocyclohexa-2,5-dien-1-yl)acetate (**4d**)

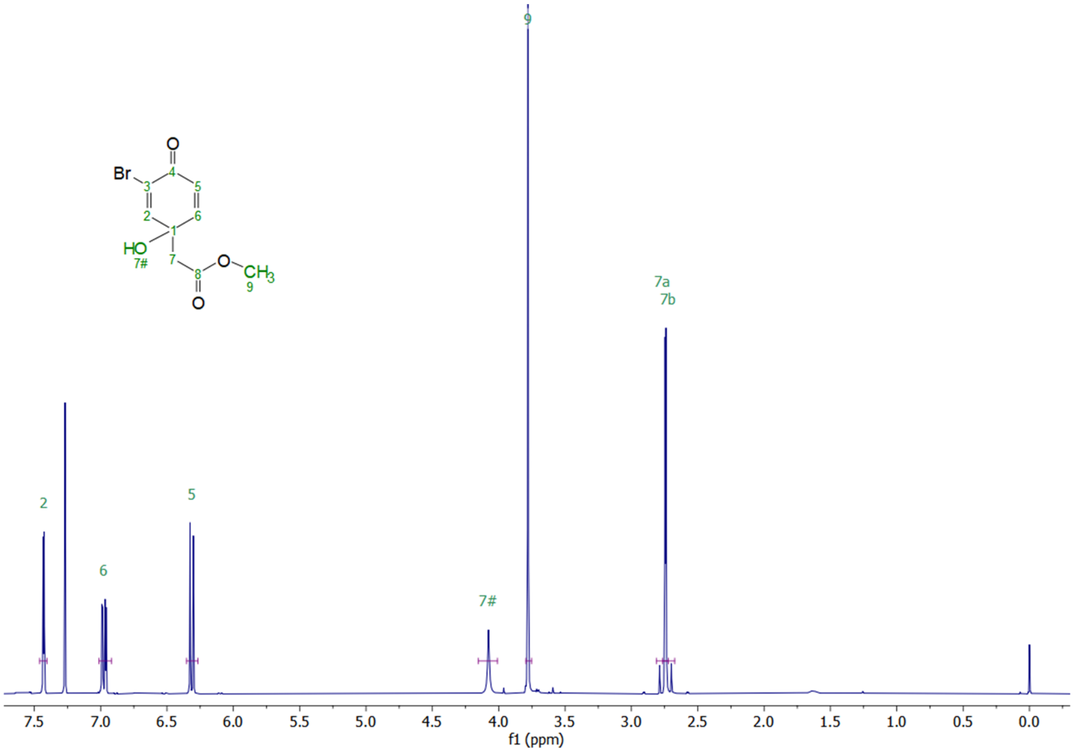

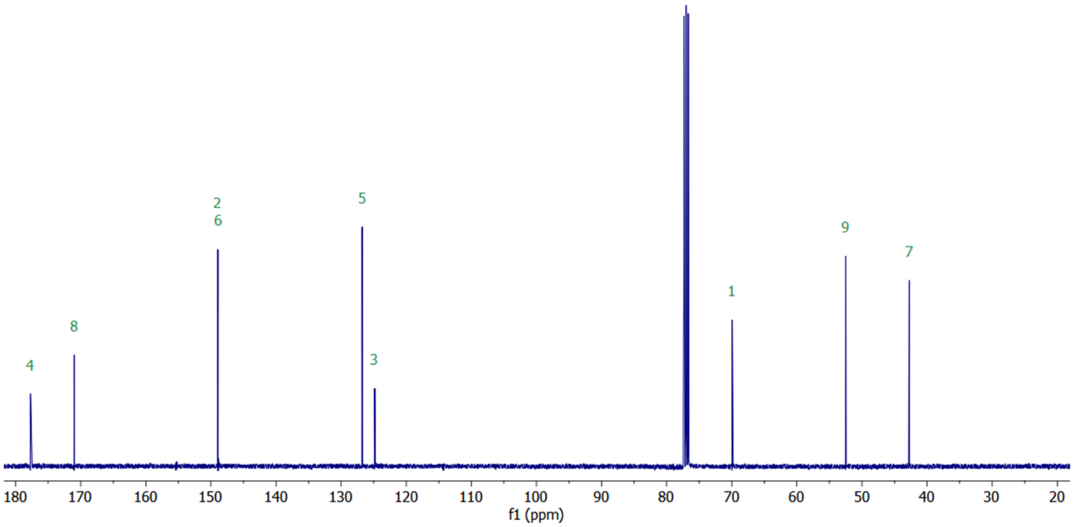

Methyl 2-(3,5-dichloro-1-hydroxy-4-oxocyclohexa-2,5-dien-1-yl)acetate (**4e**)

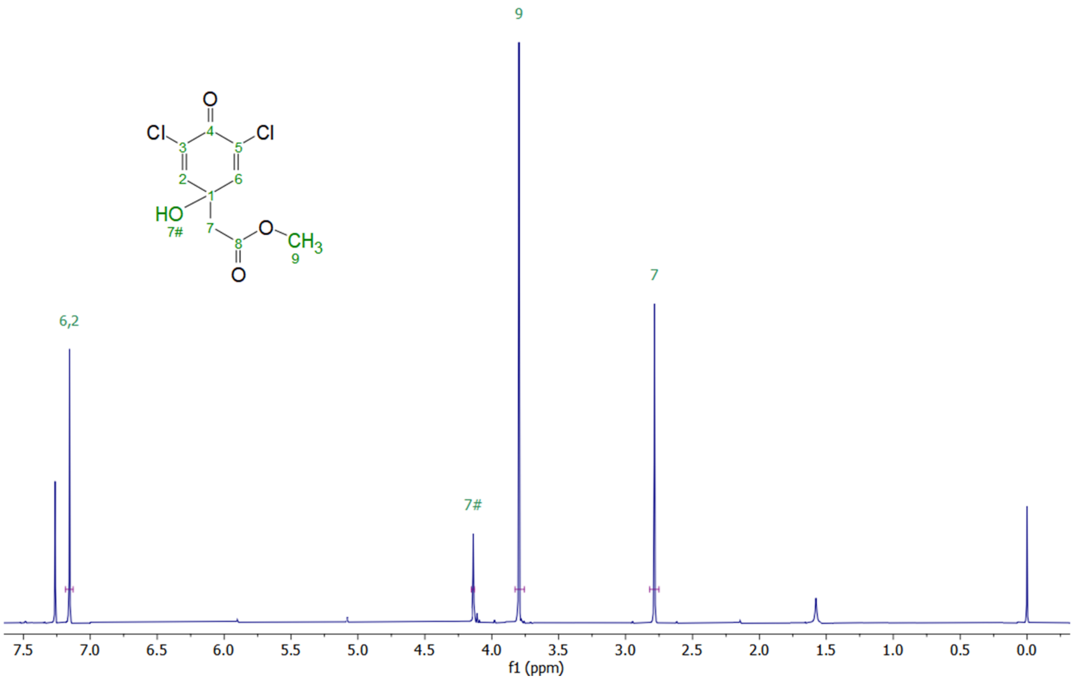

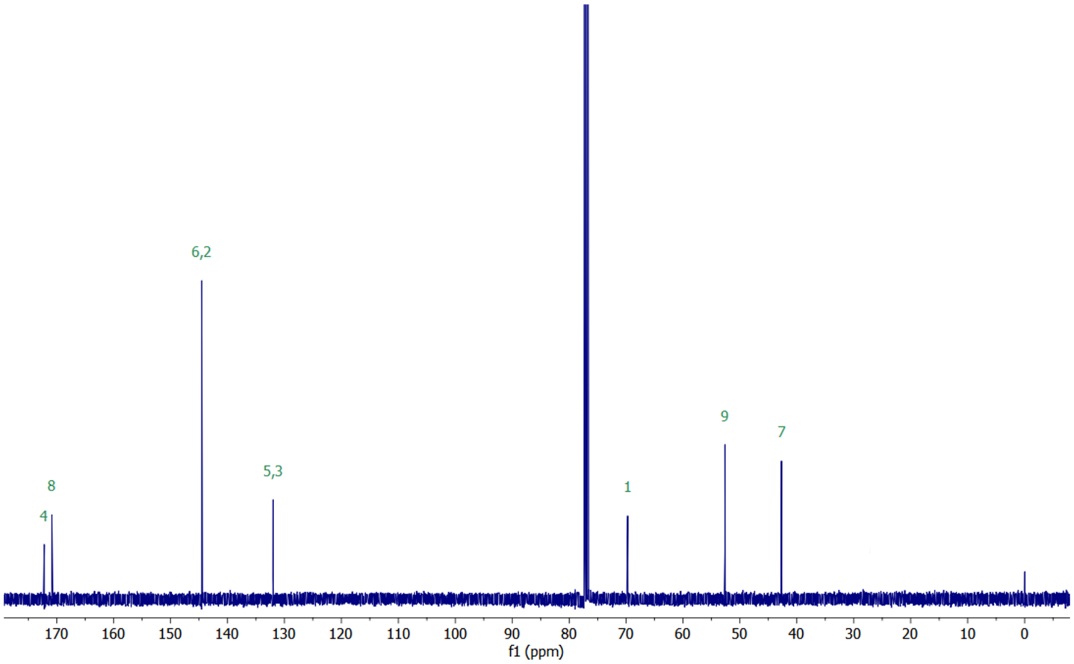

Methyl 2-(3,5-dibromo-1-hydroxy-4-oxocyclohexa-2,5-dien-1-yl)acetate (**4f**)

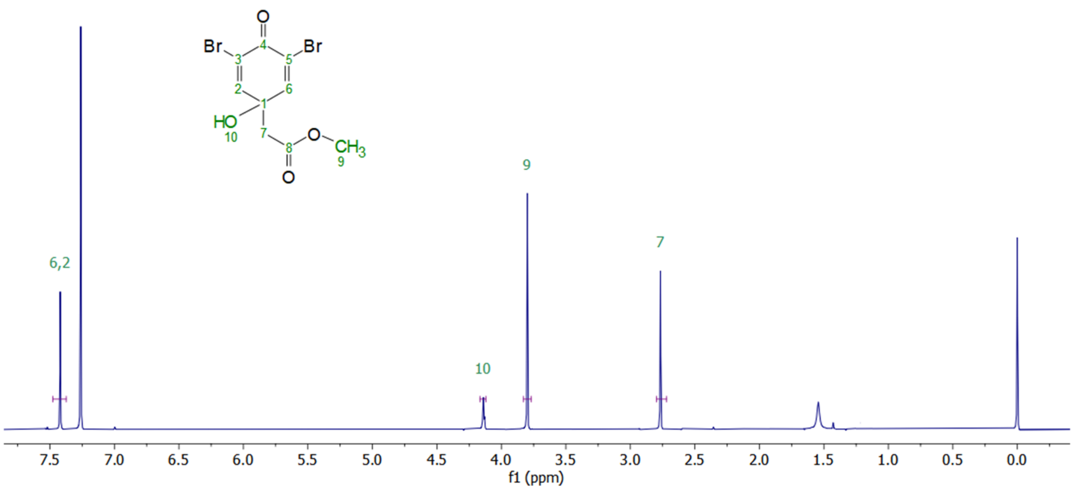

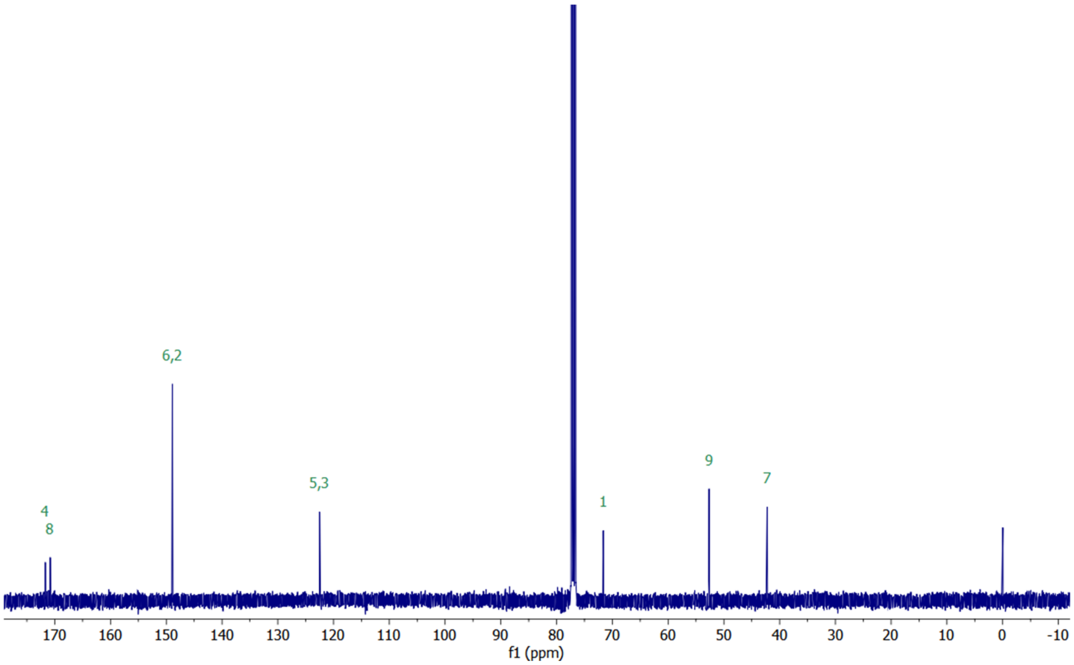

Methyl 3-(1-hydroxy-4-oxocyclohexa-2,5-dien-1-yl)propanoate (**4g**)

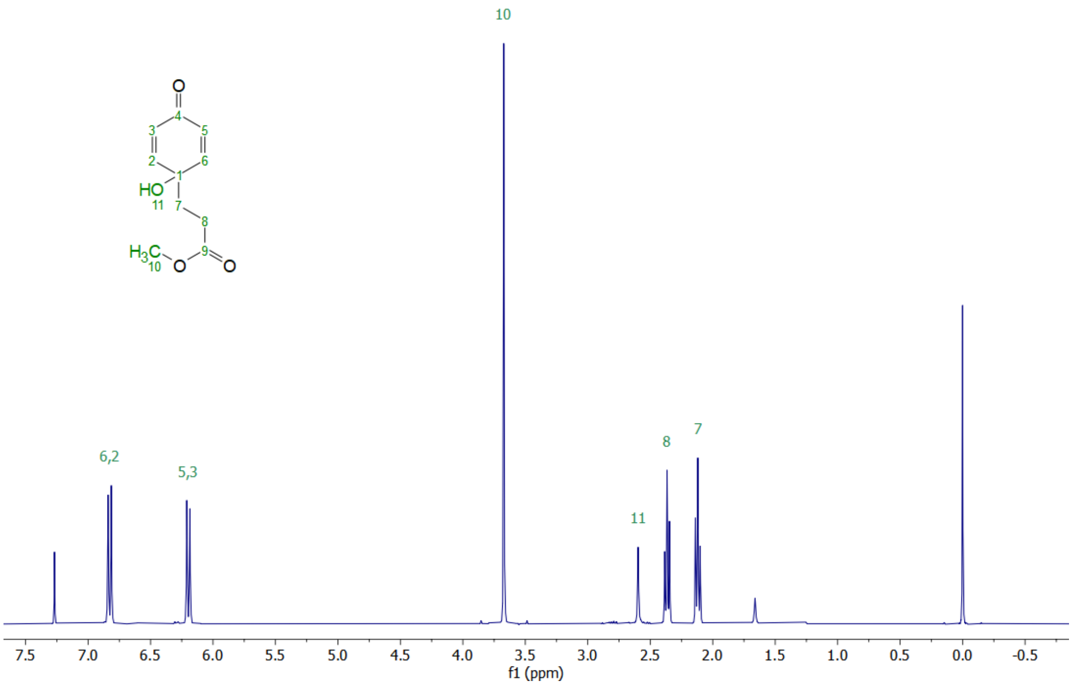

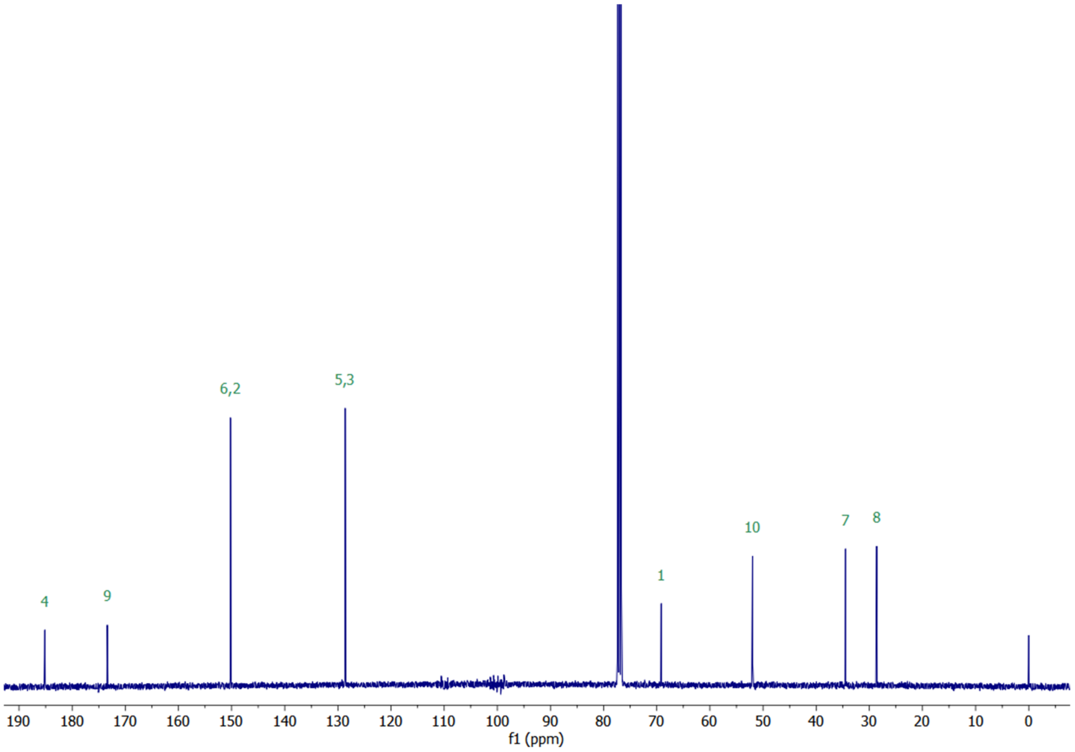

1-Oxaspiro[4.5]deca-6,9-diene-2,8-dione (**4h**)

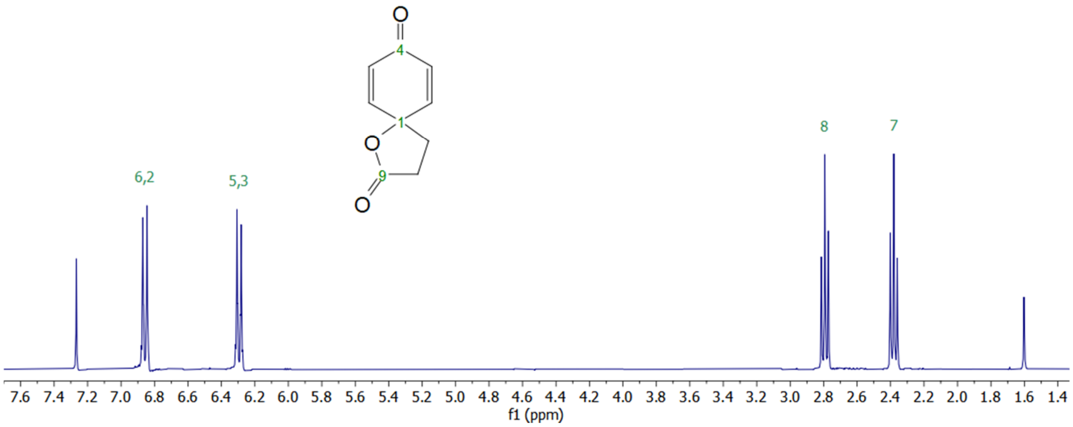

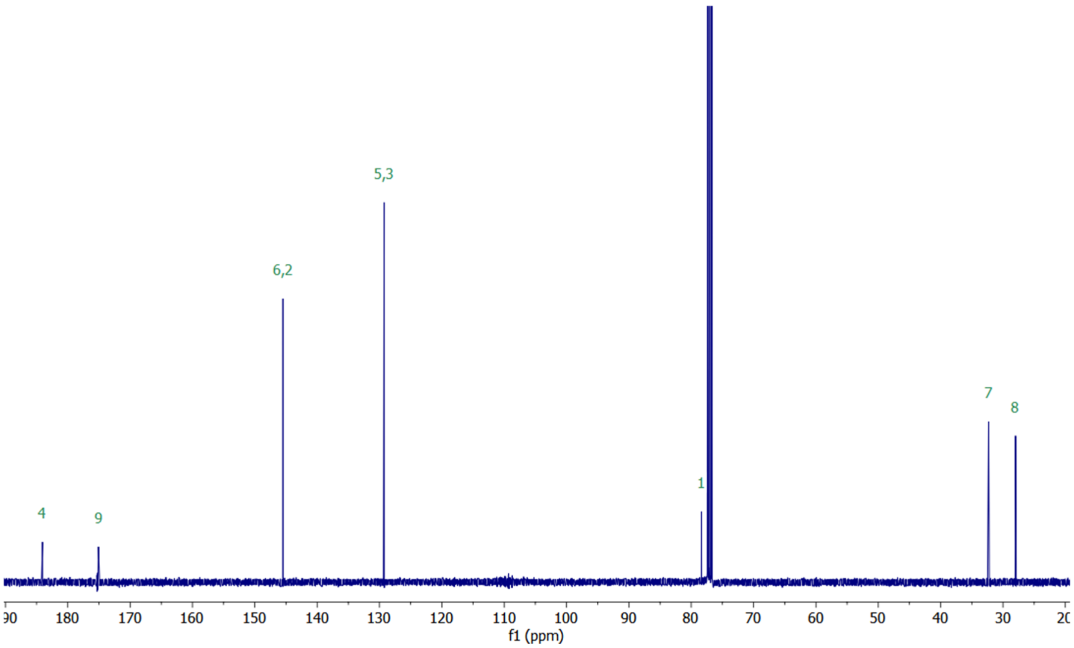

*Methyl (2E)-3-(1-hydroxy-4-oxocyclohexa-2,5-dien-1-yl)prop-2-enoate (**4i**)*

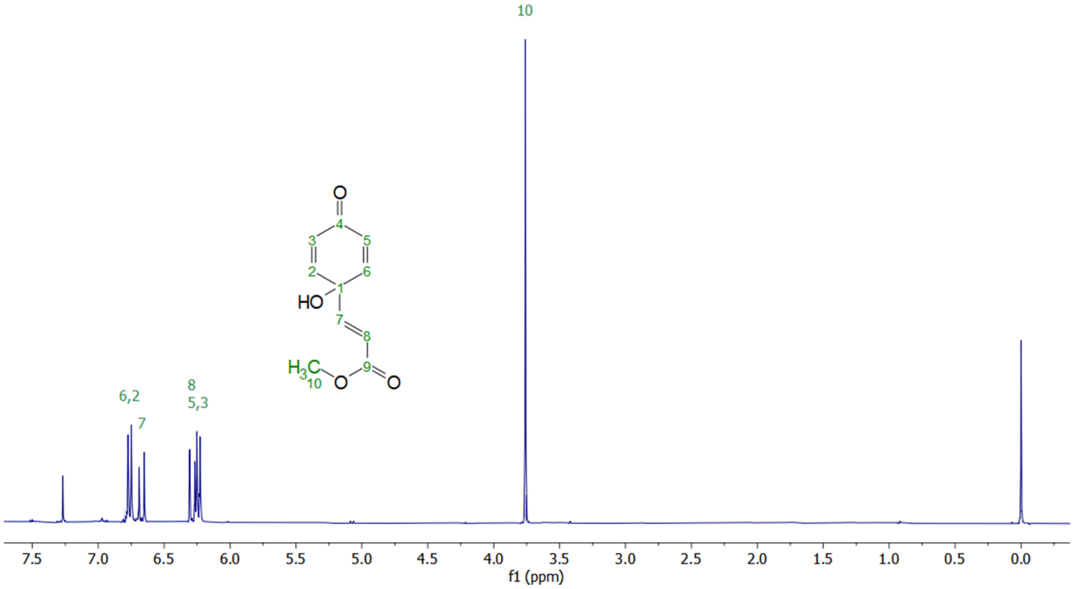

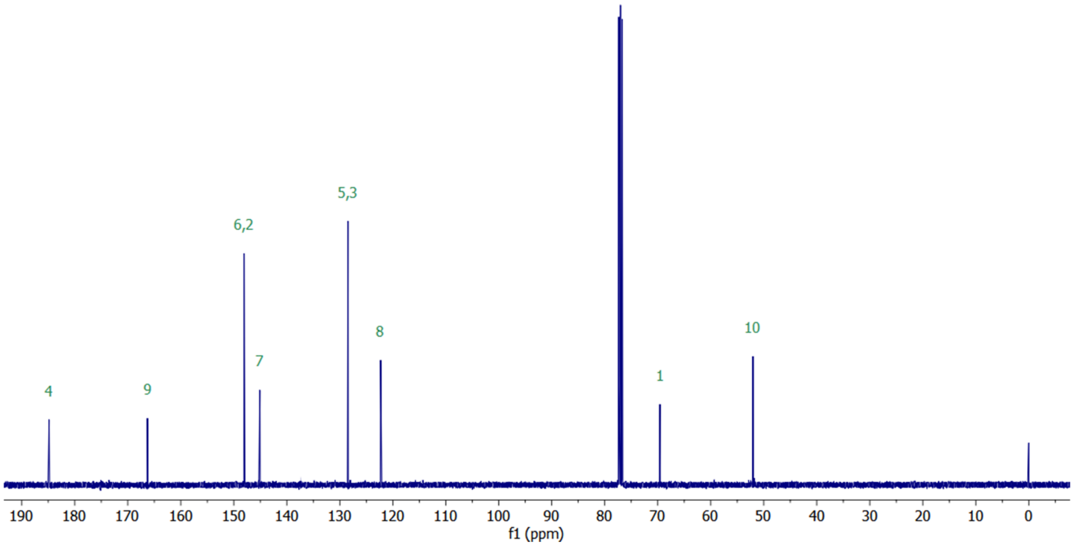

2-Benzyl-1,4-dihydronaphthalene-1,4-dione (**5c**)

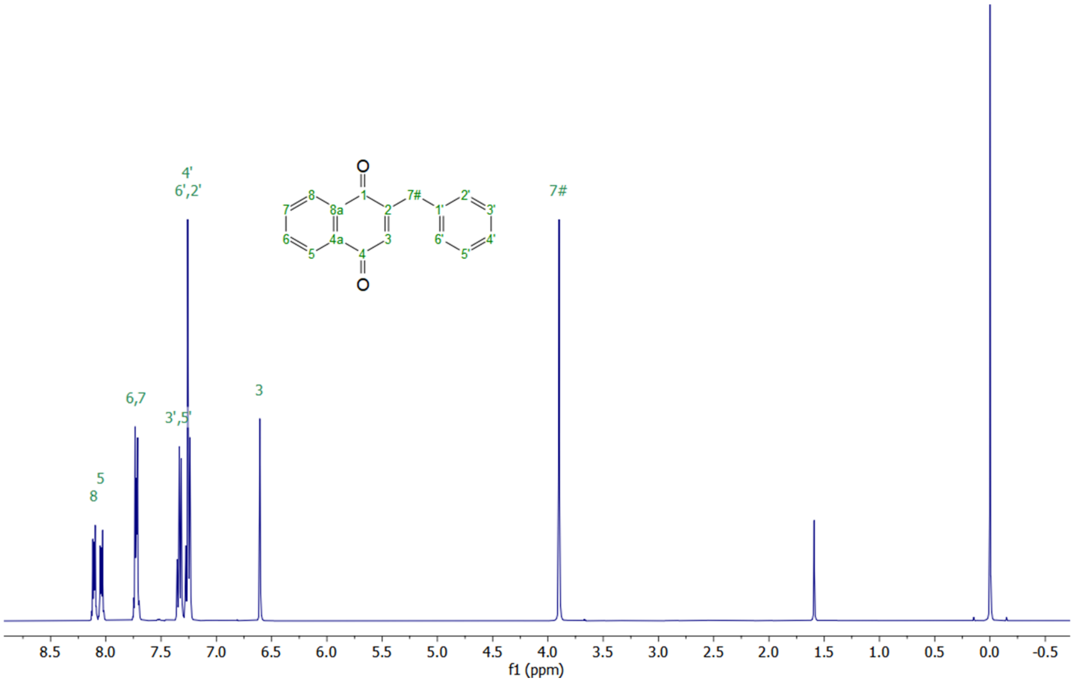

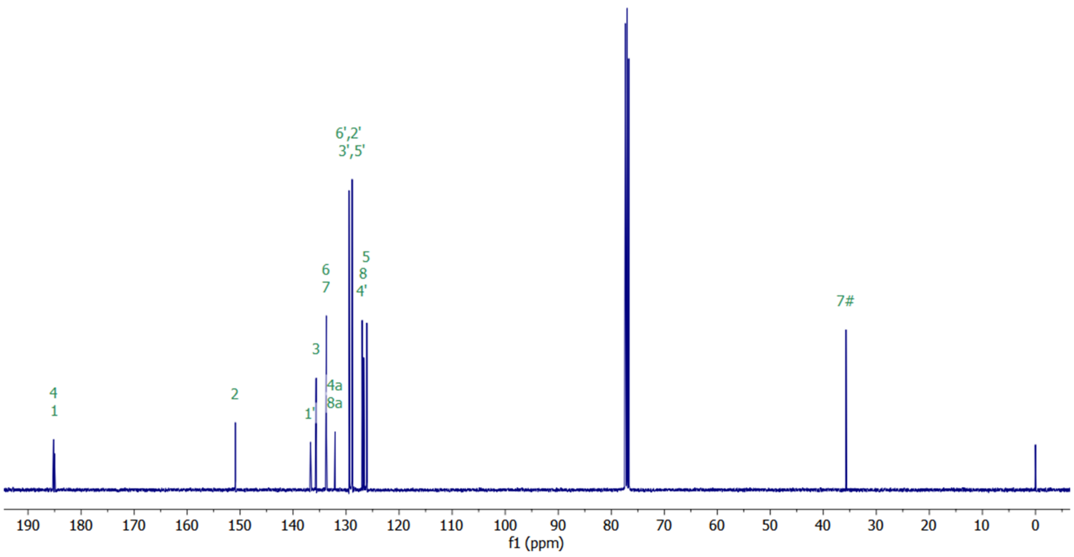

6-Fluoro-1,4-dihydronaphthalene-1,4-dione (**5d**)

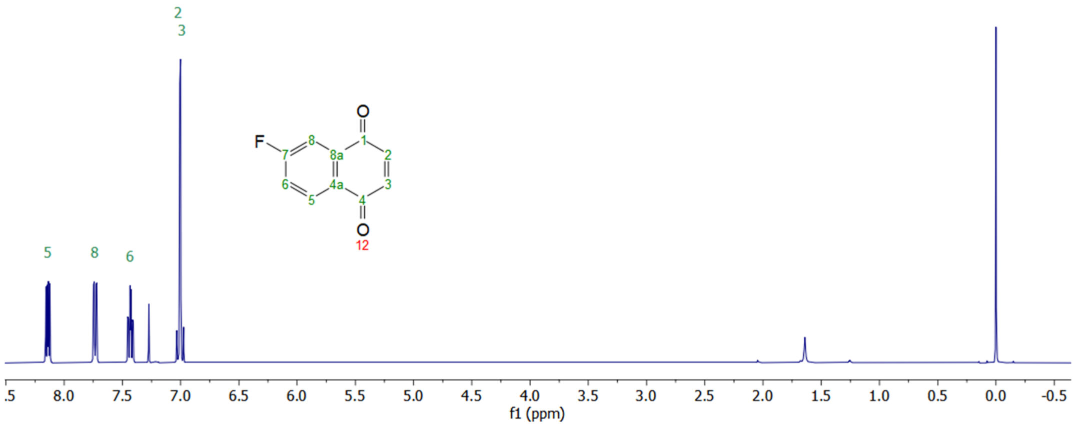

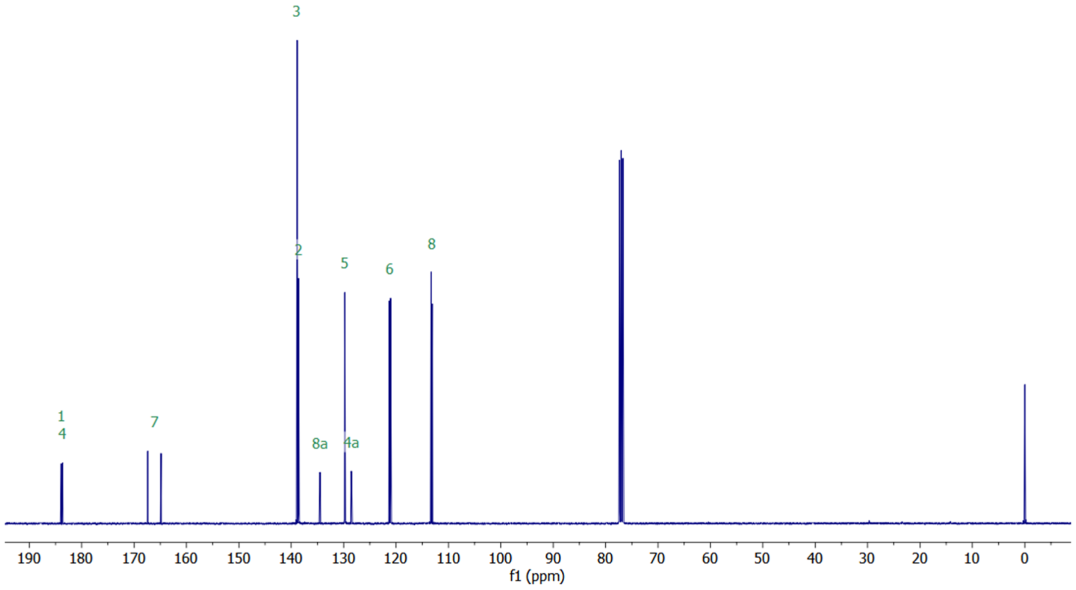

6,7-Difluoro-1,4-dihydronaphthalene-1,4-dione (**5e**)

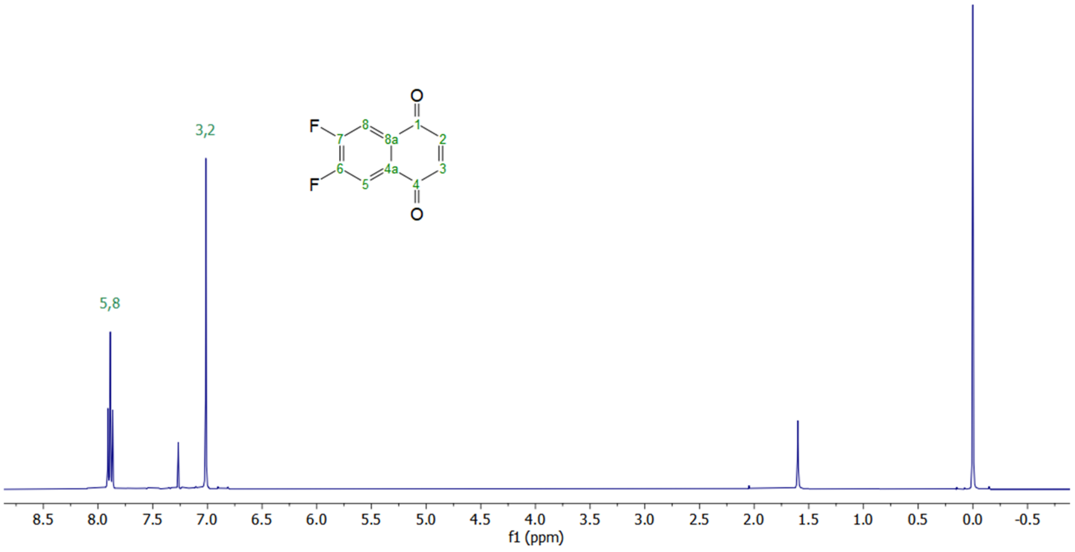

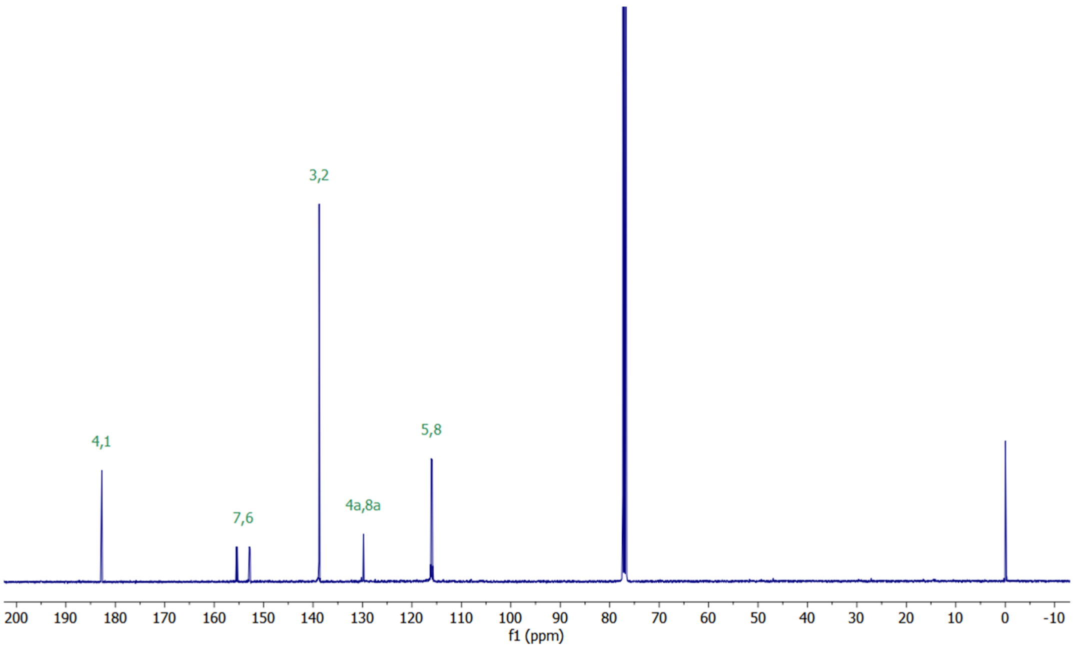

Supplement: Supplementary file 1 [file molecules-27-06559-s001.zip › molecules-1923629-supplementary.pdf]
